# Label-Free Protein Analysis by Pyro-Electrohydrodynamic Jet Printing of Gold Nanoparticles

**DOI:** 10.3389/fbioe.2022.817736

**Published:** 2022-02-22

**Authors:** Veronica Vespini, Simonetta Grilli, Pietro Ferraro, Romina Rega, Heidi Ottevaere, Yunfeng Nie, Pellegrino Musto, Marianna Pannico

**Affiliations:** ^1^ National Research Council of Italy (CNR-ISASI), Institute of Applied Sciences and Intelligent Systems, Pozzuoli, Italy; ^2^ Vrije University of Brussels Pleinlaan, Brussels, Belgium; ^3^ Composites and Biomaterials, National Research Council of Italy (CNR-IPCB), Institute for Polymers, Pozzuoli, Italy

**Keywords:** SERS spectroscopy, sensors, jet printing, colloids, plasmonic

## Abstract

The pyro-electrohydrodynamic jet (p-jet) printing technology has been used for the fabrication of confined assemblies of gold nanoparticles with a round shape and a diameter ranging between 100 and 200 μm. The surface-enhanced Raman spectroscopy (SERS) performance of the p-jet substrate was evaluated by using Rhodamine 6G (R6G) as a reference. The results demonstrate that this kind of SERS substrate exhibits strong plasmonic effects and a significant reproducibility of the signal with a coefficient of variation below 15%. We tested the signal behavior also in case of the bovine serum albumin (BSA) as a model analyte, to demonstrate the affinity with biomolecules. Strong SERS activity was measured also for BSA across the whole spot area. The spectral patterns collected in different locations of the sensing area were highly reproducible. This observation was substantiated by multivariate analysis of the imaging datasets and opens the route towards a potential application of this kind of SERS substrate in biosensing.

## 1 Introduction

Surface-enhanced Raman spectroscopy (SERS) is a versatile analytical technique widely used for molecular detection and for the investigation of a great variety of chemical and biological samples (Aroca, 2006; Le Ru and Etchegoin, 2008). Among the numerous advantages of this technique are the excellent sensitivity, the fingerprint character of the spectrum, which allows identification and multiplex analysis, and the sampling flexibility (from macro to submicro measurements, remote sampling *via* fiber optics) (Schlucker, 2011). In real-world applications, the major drawback is related to the non-uniform response of 2D SERS sensors, i.e., those obtained by deposition of colloidal solutions onto suitable substrates (Schlucker, 2011; Fornasaro et al., 2020). The effect is due to the inhomogeneous distribution of the nanoparticles across the sensing surface, which generally results after drying. Attempts to alleviate this issue mostly involve the chemical modification (derivatization) of the substrate surface, but so far, limited success has been achieved (Wang and Kong, 2015; Fornasaro et al., 2020). The problem is particularly relevant in quantitative analysis where spatial and temporal consistency of the signal is mandatory (Fornasaro et al., 2020). Strictly related to the non-uniform SERS activity of 2D sensors is the undersampling problem (Shin and Chung, 2013): in most experimental setups, spectral collection is made through a microscope objective, which irradiates (and collects signals from) an area of up to 200 µm in diameter. Thus, the spectrum is poorly representative of the whole sample (drop-casting produces footprints between 0.5 and 5.0 mm in diameter), and the SERS signals display a large spatial variability. To minimize this problem, several strategies have been proposed, each with its own disadvantages: 1) collecting and averaging spectra at many different points, which is a lengthy and cumbersome procedure, unsuitable for high-throughput analyses; 2) rotation of the sample during spectral collection, which requires specialized accessories and produces low-quality spectra; and 3) simultaneous wide-area illumination (WAI), also requiring specific setups and mainly used in the single-frequency collection mode (McGoverin et al., 2008; Strachan et al., 2010; Shin and Chung, 2013). We propose an alternative approach to the undersampling problem consisting in the fabrication of a sensing area whose shape and dimensions conform to those of the laser spot, thus allowing the whole deposition to be sampled in a single acquisition (Pannico et al., 2020). The confinement of the plasmonic nanostructure within an area of precisely controlled size and shape provides the additional advantage of increasing the nanoparticle density, which enhances the SERS sensitivity. The micro-confinement of plasmonic nanoobjects has been approached in several ways, among which are electrospinning (Zhang et al., 2012), self-assembly (García-Lojo et al., 2019; Lin et al., 2019), and electro-deposition combined with lithography (Lee et al., 2019).

Recently, inkjet printing has been developed into an effective method for fabricating micro-/nanostructure arrays as this type of printing can simply drop the ink solution in a fixed volume onto a set position (Lee et al., 2005; Yakovlev et al., 2016). Both Ag and Au nanoparticles have been printed on cellulose paper or silicon wafers for SERS purposes (Yu and White, 2010; Yang et al., 2015). Even though inkjet printing exhibits advantages in terms of controllability, still challenges remain in terms of nozzle clogging with consequent requirements of ink mixing solutions for changing sample viscosity and/or surface tension.

Here, we present an innovative SERS substrate achieved through the p-jet printing technique. In particular, we propose a significant step forward compared to the proof-of-concept published in Pannico et al. (2020) with a top-down jetting modality and with a micro-engineered loading support. This configuration produces a stable meniscus profile independent from the load volume, allowing us to obtain spots of gold nanoparticles with high reproducibility in terms of both size and SERS signal, thus significantly increasing the statistical validity of this innovative SERS substrate.

The p-jet printing is an attractive technology for fabricating micro-scale patterns due to its advantages in terms of low cost, efficient use of materials, and waste reduction (Ferraro et al., 2010). By precisely controlling the printing droplets, one can print high-resolution patterns with versatile materials directly on substrate thanks to an electric field generated by the pyroelectric effect from lithium niobate (LN) crystal subjected to a temperature gradient. Additionally, the p-jet allows us to print the colloidal solution of nanoparticles through a non-contact modality avoiding nozzles and external electrodes, thus overcoming the clogging-related issues and the need for mixing buffers (Nie and Kumacheva, 2008).

We characterize here the SERS signal of round-shaped spots, with a diameter in the range of 150–200 μm, made of an assembly of gold nanoparticles, first with R6G and then with a typical test protein, BSA. In fact, the detection of proteins in complex mixtures, with or without preliminary separation, is a central problem in biochemical research (Bergveld, 1991; Westermeier and Marouga, 2005; Han et al., 2009) (Mann et al., 2001; Domon and Aebersold, 2006) (Ladokhin, 2006; Hillger et al., 2007), and SERS spectroscopy is the object of interest for this kind of application (Han et al., 2008; Han et al., 2009; Foti et al., 2018). When the measurement is carried out in colloidal solutions, the sensitivity is lowered by dilution and the spectrum shows a spatial variability, possibly reflecting multiple binding sites and/or conformational rearrangements around the binding site (Szekeres and Kneipp, 2018; Kuhar et al., 2021). The inconsistency of the SERS pattern complicates the use of the method for identification and quantification purposes. We demonstrate here how this innovative SERS substrate displays a substantial SERS activity and, above all, a good reproducibility of the spectrum across the whole sensing area, as confirmed by multivariate analysis of the hyperspectral data sets. These features open the perspective of multiplex analysis and quali/quantitative assays in complex biological mixtures.

## 2 Materials and Methods

### 2.1 Materials

Trisodium citrate (TC), gold (III) chloride trihydrate (HAuCl_4_·3H_2_O), ascorbic acid (AA), sodium borohydride (NaBH_4_), cetyltrimethylammonium bromide (CTAB), rhodamine 6G (R6G), and bovine serum albumin (BSA) have been supplied by Sigma Aldrich (Italy) and used as received. Milli-Q water was used for the preparation of gold nanoparticles. LN, a rhombohedral crystal belonging to the point group 3 m, was bought from Crystal Technology Inc. in the form of both sides polished, 500 μm thick, z-cut, and 3-inch diameter. The wafer was cut into square samples 2 × 2 cm^2^ in size by a standard diamond saw. The deposition slide was a coverslip, bought from Sigma Aldrich, consisting of an untreated glass substrate 24 × 60 mm in size and 0.2 mm thick.

### 2.2 Fabrication of the P-jet SERS Substrate

Gold nanoparticles (AuNPs) were synthesized in aqueous solution by using the seed-mediated growth method as described in a previous work (Pannico et al., 2020). A SERS substrate was obtained by deposition of the colloidal solution on a glass substrate by pyro-electrohydrodynamic jet-printing technique.

#### 2.2.1 Pyroelectric Effect

The spontaneous polarization *P*_s_ of the LN crystal changes according to ∆*P*_s_ = *p* ∆*T*, where *p* is the pyroelectric coefficient and *ΔT* is the temperature variation (*p* = −8.3 × 10^–5^C m^−2^ K^−1^ at *T* = 298 K). At thermal equilibrium, the spontaneous polarization of the crystal is completely screened by the external charges accumulated on the crystal surfaces, and no electric field is present. When the crystal is stimulated through a temperature variation, an uncompensated surface charge density = ∆*P*_s_ and electric potential difference $\Delta U{∼}^{\sigma d/\varepsilon o\varepsilon }$ appear on the crystal surfaces (here, *d* is crystal thickness and $\varepsilon_{o}$ and ε are dielectric constants of vacuum and of the crystal, respectively). This potential difference can reach 10^4^ V for millimeter-sized crystal thickness and *ΔT* of about 10 K.

#### 2.2.2 Thermal Stimulation

The thermal stimulation is insured using a tungsten wire-based heater onto lower side of the LN crystal, able to apply a temperature gradient on LN and to generate the pyroelectric effect. The heat transfer device has been fabricated in lab workshop with a tungsten wire having 300 μm diameter and a pointed shape. The heater is powered by a high-current power supply that is modulated with a 5-V transistor–transistor logic (TTL) signal generated by a conventional signal generator. The “ON time” represents the time lapse in which heating current passes through the heater, “Total time” is the period of thermal stimulus, and “OFF time” = “Total time” − “ON time” is the time during which the pyroelectric layer comes into thermal equilibrium with the environment. The electrical circuit is set by enabling the power supply (Lab. Grade Switching Mode Power Supply HCS-3300), and a waveform generator (33220A Function/Arbitrary Waveform Generator, 20 MHz) is utilized to set a step function with assigned on and off times. In addition, the system includes a fan blowing air to the cooling structure of pyroelectric stage, LiNbO3 crystal. The aim of the efficient cooling is to enable faster heating cycles for the dispensing. The electrical and thermal characteristics of the investigated heater is presented in a table, where *R*, *I*_ON_, *P*_ON_, *T*_ON_, and *P*_MEAN_ are heater resistance, heating pulse current, heating pulse power, heating pulse temperature, and mean power of periodic heating pulses, respectively, as shown in Table 1.

**TABLE 1:** Electrical and thermal characteristics of the heater.

Heater	R (Ω)	*I*_ON_ (A)	ON time (s)	OFF time (s)	*P*_ON_ (W)	*P*_MEAN_ (W)	*T*_ON_ (°C)
Tungsten wire	0.2	8.5	3.5	56.5	14.5	0.85	120

#### 2.2.3 Loading Support Configuration

Loading support is made of a micro-orifice in PMMA HESAglas using a hot molding approach; the upper diameter is around 5 mm while the bottom orifice is around 1 mm. A mother drop (around 10 μl) of sample is loaded manually by a standard pipette into the engineered micro-orifice of the loading support, and a stable liquid meniscus emerged on the opposite side. The aim is to achieve a liquid meniscus and, consequently, a more accumulated charge on its apex to shorten the diameter of the ejected droplet and improve its repeatability. The surface smoothness has been also measured to be within the sub-micron range, demonstrating a good smoothness of the orifice. This allows to have an innovative contact-free modality as a means for generating a pointed meniscus exposed to the pyro-electric field for tiny-droplet ejection by p-jet. In addition, the system with orifice translates the loading layer in *Z* direction with a manual translation stage.

### 2.3 SERS Activity

The SERS performance was evaluated by using R6G as a reference. The p-jet SERS substrate was washed several times with Milli-Q water, dried under nitrogen flux, and then functionalized with a 100 μM water solution of R6G: 30 L of R6G solution were dropped on the substrate to fully cover the whole spots. The substrate was kept at room temperature, for a sufficient time, to allow water evaporation. Before Raman characterization, the substrate was washed again with water to remove the excess of unreacted R6G and dried under nitrogen flux. The same procedure was adopted when testing the present analytical platform for BSA detection. Thus, a 100-M water solution of BSA was deposited by drop-casting. The substrate was washed with Milli-Q water several times to remove the excess protein and dried under nitrogen flux.

### 2.4 Techniques

#### 2.4.1 Transmission Electron Microscopy

The AuNP shape and average diameter were estimated by bright-field transmission electron microscopy (TEM) performed on an FEI Tecnai G12 Spirit Twin (LaB6 source) equipped with an FEI Eagle 4K CCD camera (Eindhoven, The Netherlands) operating with an acceleration voltage of 120 kV. Samples for TEM examination were prepared by immersing a carbon-coated copper grid in the colloidal solution, followed by drying. TEM images were acquired in different sample areas and were transferred to the MATLAB computational platform (MathWorks, Natick, MA, United States ) for further processing. Statistical image analysis (SIA) was performed by the PLS/MIA toolbox (Eigenvector Research Inc. Manson, WA, United States ), running under the MATLAB environment.

#### 2.4.2 Zeta Potential Measurements

Zeta potential of the CTAB-coated nanoparticles was measured with a Malvern Zetasizer nano series (Malvern Instruments Ltd. Malvern, United Kingdom). For *Z*-potential measurements, a capillary cell type ZEN 1020 was employed. Zeta potential measurements were done before and after the centrifugation cycles to verify the AuNP solution stability. The reported value is the estimated mean of three measurements.

#### 2.4.3 UV-VIS Spectroscopy

The optical properties of AuNPs and their molar concentration were measured by a UV spectrophotometer equipped with single monochromator (V−570 from Jasco, Easton, United States ). Absorption spectra of the AuNP colloids were collected using a 1.00-cm quartz cell with a scan speed of 400 nm/min in the wavelength range from 300 to 800 nm. For the quantitative analysis of the gold amount in the AuNP solution, a set of six standards were obtained by diluting the as-prepared gold nanoparticle solution batch. Complete reduction from Au(III) to Au (0) was assumed.

#### 2.4.4 Raman Spectroscopy

The SERS performance of AuNP colloids was evaluated by Raman spectroscopy using R6G as a molecular probe. The Raman and SERS spectra were collected by a confocal Raman spectrometer (Labspec Aramis, from Horiba-Jobin Yvon, Edison, NJ, United States) operating with a 632 nm diode laser as the exciting source. The 180° back-scattered radiation was collected by an Olympus metallurgical objective (MPlan ×10, NA = 0.25) with confocal and slit apertures set to 400 μm; a grating with 600 grooves/mm was used throughout. The radiation was focused onto a CCD detector (Synapse Mod. 354,308) cooled at –70°C by a Peltier module. The laser power measured at the output of the objective was 1.87 mW, which resulted in a power density of 1.9 mW/μm^2^. The reference spectrum of R6G was collected on a 2 mM aqueous solution with an exposure time of 8 s. The SERS spectrum of R6G was collected after AuNP functionalization with a 100 M ethanol solution of R6G (AuNPs/R6G, *v*/*v* 1:1) with an exposure time of 3 s. Both spectra were acquired by using a quartz cuvette with a chamber volume of 700 μl (Hellma GmbH and Co., Jena, Germany).

R6G was further used to test the SERS performance by running Raman imaging measurements in the mapping mode: the p-jet SERS substrate was placed on a piezo-electrically driven microscope stage with an *x*, *y* resolution of 10 ± 0.5 nm and a *å* resolution of 15 ± 1 nm. The stage was scanned at a constant speed in the *x*–*y* plane with an 8.0-μm step size. The spectra were collected by an Olympus metallurgical objective (MPlan ×10, NA = 0.25) with a 632-nm diode laser, an exposure time of 10 s, and confocal and slit apertures set to 400 μm. The same acquisition parameters were used to carry out Raman imaging measurements on the substrate functionalized with BSA. The only difference was the step size that was settled to 5 μm. The reference BSA spectrum (bulk) was collected by an Olympus metallurgical objective (MPlan ×10, NA = 0.25) with a 632-nm diode laser, an exposure time of 15 s, and confocal and slit apertures set to 400 μm. A 500-μM BSA water solution (120 μl) was dropped on an aluminum support and let dry at room temperature before the Raman acquisition. All the collected Raman data were converted into ASCII format and transferred to the MATLAB computational platform for further processing.

## 3 Results and Discussion

### 3.1 Pyro-Electrohydrodynamic Jet System

**FIGURE 1:**
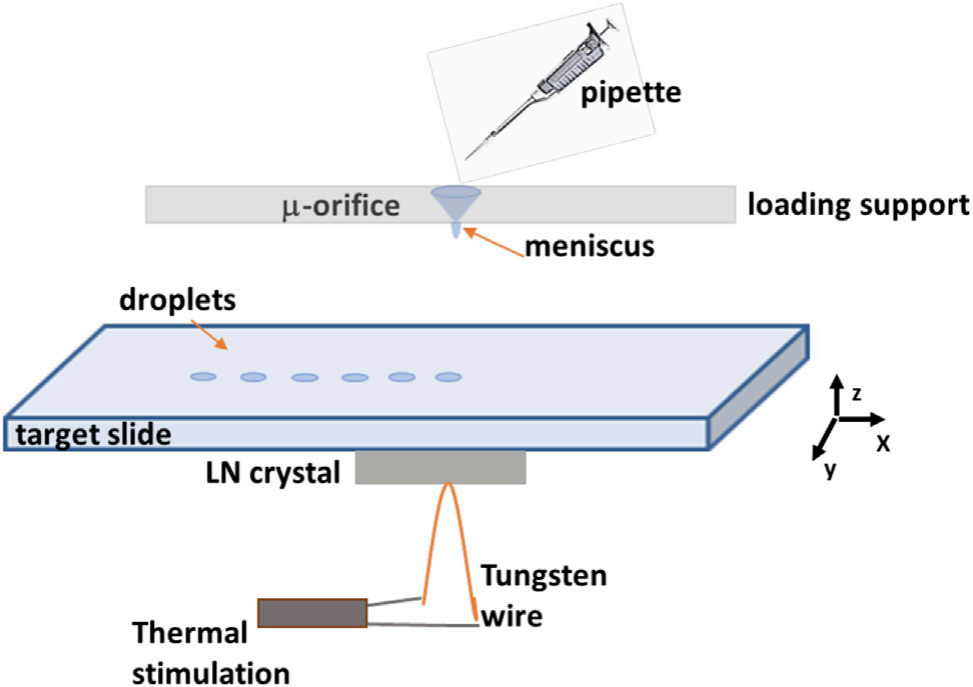
The schematic shows the p-jet configuration.

The basic principle of the p-jet printing is based on the pyroelectric effect and, therefore, on the use of ferroelectric crystals (LN) and the manipulation of the liquid samples in the presence of an electric field generated pyroelectrically. Figure 1 schematically shows the p-jet system, used here for fabricating the sensing surface. A mother drop of a solution sample containing an amount of gold nanoparticles is loaded directly in a micro-orifice of the loading support, allowing the formation of a thin liquid meniscus on the opposite side. Moreover, a standard XYZ micrometric manual translation stage is used for aligning the orifice with the central point of a piece of LN crystal, while a heating element made of tungsten wire is placed in contact with the crystal, and an electric field induced by the pyroelectric effect exerts an attractive force on the drop-meniscus deforming and generating liquid jet emission. The target slide, printing the tiny droplets, is inserted between the loading support with meniscus and the LN crystal.

**FIGURE 2:**
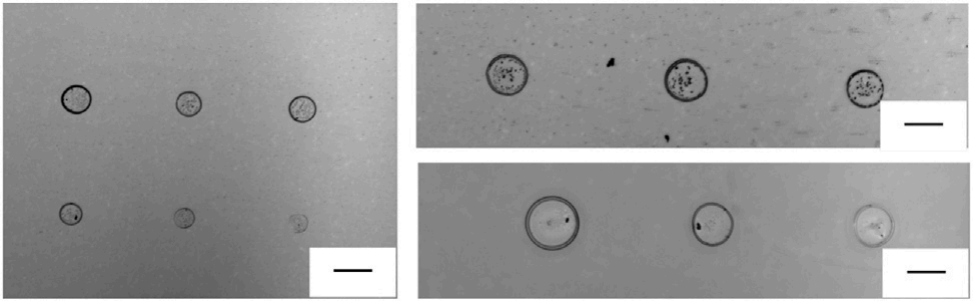
Optical images of a series of dots printed with reproducible geometry onto a conventional glass target slide (scale bar is 200 μm).

The great advantage of this technique consists in the possibility of direct printing micro-drops onto a sensing surface, used as the receiving substrate. In fact, pyro-EHD printing allows to realize high-resolution samples, keeping good precision during the printing process, and fine spatial resolution onto a very large scale could be obtained. In addition, several nanostructured plasmonic sensor could be used through pyro-EHD printing. This technology has proven to be highly repeatable with spot diameter around 200 μm as shown in Figure 2. Moreover, the *p-jet* system has been improved with respect to the previously published study; these upgrades allowed us to improve the reproducibility of the micro-patterns in terms of size/geometry as shown in the next images.

**FIGURE 3:**
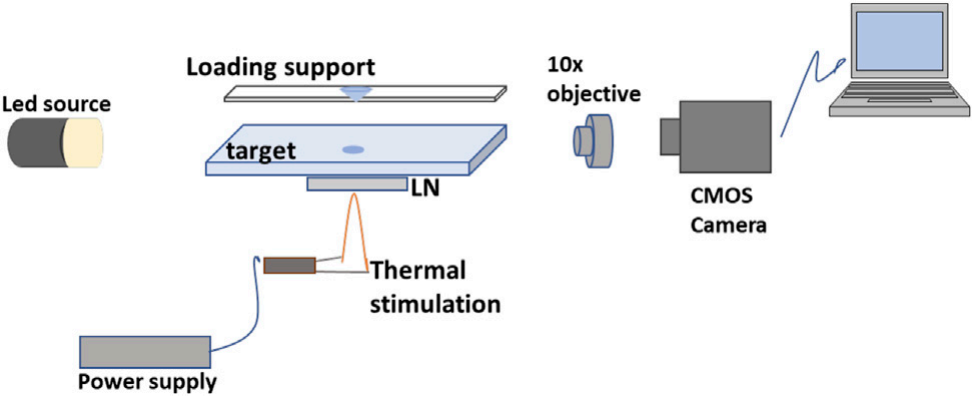
Schematic view of the p-jet system.

Figure 3 shows a traditional optical path, consisting of a collimated LED, an optical microscope objective (×10), and a high-speed CMOS camera (Motion Pro Y3-S1, pixel size of 10.85 μm2), allowing us to observe the ejection event during the activation of the pyroelectric effect. The electric field generated by the pyroelectricity of the LN crystal causes the accumulation of charge on the free surface of the mother droplet through the electrostatic effect. The charge density is high enough to cause the well-known Coulomb repulsion effect (Collins et al., 2008; Gennari et al., 2015), which deforms the meniscus of the mother droplet into a so-called Taylor cone. When the electric field exceeds the surface tension of the liquid, tiny droplets are ejected from the apex of the meniscus (Hartman et al., 1999). The volume of sub-droplets produced by this p-jet system is in the range of 100 pL and are deposited on a simple microscope glass previously cleaned. After the first spot, we translate the deposition glass slide and generate a new point of a colloidal solution containing gold nanoparticles under certain spatial constraints.

### 3.2 Colloid Characterization

**FIGURE 4:**
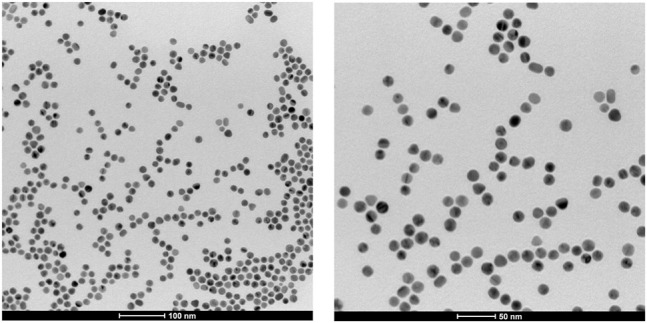
Transmission electron microscopy (TEM) micrographs of gold nanoparticles at different magnifications.

According to our previous work (Pannico et al., 2018), the shape and shape distribution of the AuNPs were evaluated from SIA. Ten TEM images (nanoparticle population of about 900 units) were used for the analysis. In Figure 4, two representative TEM micrographs of the AuNPs are shown, at different magnifications: it was found that 98% of the population is characterized by a spherical shape (*R* ≥ 0.8); it exhibits an average diameter of 16 ± 3 nm and a unimodal, Gaussian-like size distribution (*cf* Supplementary Figure S1). Only 2% of the total nanoparticle population was characterized by a rod-like shape (*R* ≤ 0.6). In the whole set of TEM images, no particle aggregation was apparent. In fact, the CTAB surfactant adsorbs onto the AuNP metal surfaces forming a positively charged self-assembling bilayer (Suh and Moskovits, 1986) as confirmed by the zeta potential value of +43 ± 3 mV. This value hardly changed, even after centrifugation and re-dispersion in Milli-Q water (+40 ± 2 mV), confirming the stability of the bilayer structure (Nikoobakht and El-Sayed, 2001; Sau and Murphy, 2005; Das et al., 2008).

By UV-VIS measurements, the AuNP colloids exhibit a strong local surface plasmon resonance (LSPR) band around 526 nm with a narrow shape, which, in accordance with the SIA results, is indicative of a unimodal distribution of particle sizes (see Supplementary Figure S1, Supplementary Material S1). The Au molar concentration in the colloidal solution was evaluated by using a calibration curve. The Au molar concentration was estimated to be 2 mM. According to our previous work (Pannico et al., 2018), by coupling the TEM geometrical parameters (size and shape) and the UV-VIS results [(Au)], the concentration of the colloid solution in terms of nanospheres was estimated to be 17 nM. This AuNP batch was used for the fabrication of the p-jet SERS substrate. The SERS performance of the synthesized AuNPs was evaluated in terms of absolute enhancement factor (EF). The EF was estimated by comparing the spontaneous and the SERS spectra of R6G:

$$EF=\left(I_{SERS\;\;}\times \;N_{REF}\right)/\left(I_{REF}\;\times \;N_{SERS}\right)$$

where *I*_SERS_ is the integrated area of a specific SERS signal (at 1,510 cm^−1^) and *I*_REF_ is the integrated area of the corresponding Raman signal (at 1,519 cm^−1^), both normalized for the exposure time. Analogously, *N*_SERS_ and *N*_REF_ represent the number of molecules contributing to the SERS and the Raman signal, respectively. More details on the EF calculation are reported in our previous work (Sjöberg et al., 2014). The EF value of the AuNP colloid was 2.5 × 10^5^, a figure suitable for hypersensitive analytical applications.

### 3.3 SERS Response With R6G

**FIGURE 5:**
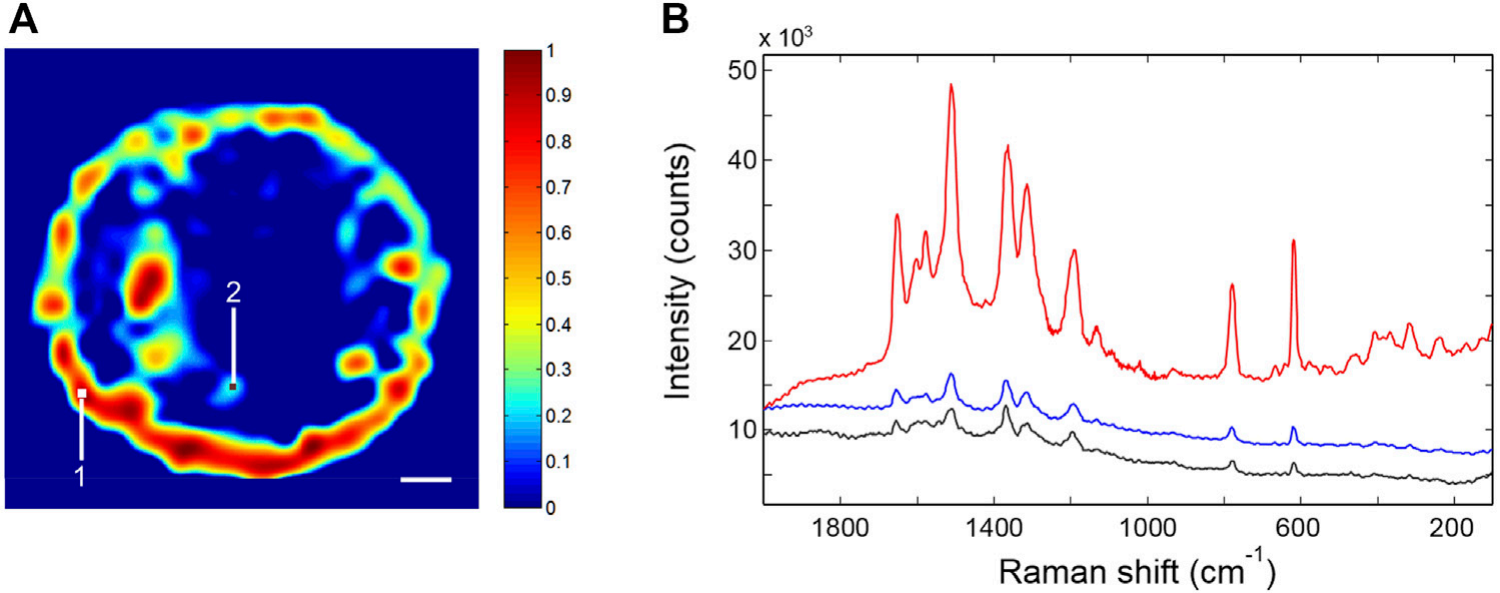
**(A)** Typical Raman image of the SERS p-jet spot functionalized with R6G. The scale bar corresponds to 20 μm. **(B)** SERS spectra collected in points one (red trace) and two (black trace) of the plasmonic surface (locations labeled in Figure 5A); SERS spectrum averaged over the whole dataset (blue trace).

The SERS activity was investigated by Raman imaging experiments. The first analysis was performed by use of R6G as molecular reporter, owing to its exceptional inelastic scattering and the well-characterized adsorbate structure (Qiu et al., 2006; Le Ru et al., 2007). After R6G surface functionalization, an area of 200 × 200 μm was mapped with a spatial resolution of 8.0 μm. Five spots were mapped, and a representative Raman image, reconstructed by considering the intensity of the R6G peak at 1,510 cm^−1^, is reported in Figure 5A. The SERS spectrum averaged over the whole dataset and two spectra collected in different points of the sensing area (labeled one and two in the Raman image) are compared in Figure 5B.

**FIGURE 6:**
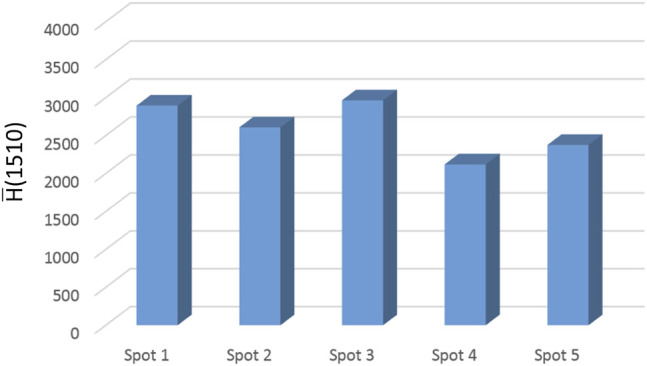
The intensity of the 1,510-cm^−1^ peak of R6G evaluated on the SERS spectrum averaged over the whole sensing area. The $\bar{H}\left(1510\right)$ value has been determined on five distinct SERS spots.

The SERS activity is strong, albeit the signal distribution is not fully homogeneous. The intensity is larger around the spot boundary (a coffee-ring effect), but regions of intense signaling are also found inside the active surface. The R6G spectrum is highly reproducible. The intensity of the 1,510 cm^−1^ peak, averaged over the whole sensing area, is represented in Figure 6 for the five analyzed SERS spots. It is found that the adopted fabrication method consistently affords the confinement of the nanoparticle assembly in a round-shaped disc having a size in the range 100–150 μm; this nanostructured pattern displays a well-detectable and reproducible SERS response [ $\bar{H}\left(1510\right)=2592\;\pm 353]$ .

The distribution of the nanospheres within the sensing area can be made more homogeneous to alleviate under sampling issues. A promising approach to achieve this result, actively investigated in our laboratories, is the derivatization of the substrate surface. The enhancement factor of this SERS substrate was evaluated by comparing the Raman and SERS spectra of R6G. The reference Raman spectrum was collected in the solution. The number of scattering molecules in the sampling volume was estimated to be 2.73 × 10^9^. We used the integrated peak area at 1,510 and 1,519 cm^−1^ for SERS and Raman, respectively. The EF was evaluated in the region of the highest SERS activity (red trace in Figure 5B), due to the inhomogeneous distribution of the nanoparticles in the sensing area, which prevents a reliable averaging of the surface coverage; it amounts to 3.0 × 10^4^. More details on the EF calculations are reported in the Supplementary Material. The EF of the SERS substrate is lower than that measured on the colloidal solution, which is representative of the intrinsic nanoparticle activity. In our opinion, the effect can be ascribed to the incomplete coverage of the plasmonic surface due to a transfer/rinsing protocol yet to be optimized. Table 2 shows the results obtained for the five p-jet spots in terms of size and SERS activity. In both cases, a coefficient of variation much below than 15% was achieved.

**TABLE 2:** Measurement results for the five p-jet spot: mean spots diameter, average SERS intensity, and coefficient of variation values.

Parameter	Mean (±SD)	Coefficient of variation (CV)
Diameter (μm)	109 (±8)	7
Intensity (a.u.)	2,592 (±353)	13

The results clearly show the high level of reproducibility both in terms of spot diameter, with a coefficient of variation less than 10%, and in terms of SERS response with a coefficient of variation less than 15%. Therefore, the step forward in the p-jet configuration presented in this work is of fundamental importance for increasing the statistical validity of the innovative p-jet SERS substrate. Compared to Pannico et al. (2020), where we demonstrated for the first time the proof of concept of a completely new p-jet SERS substrate, here we refine the p-jet configuration demonstrating the statistical validity of the technique, thanks to the high reproducibility reported in Table 2, a fundamental aspect for any analytical application.

### 3.4 SERS Response With BSA

**FIGURE 7:**
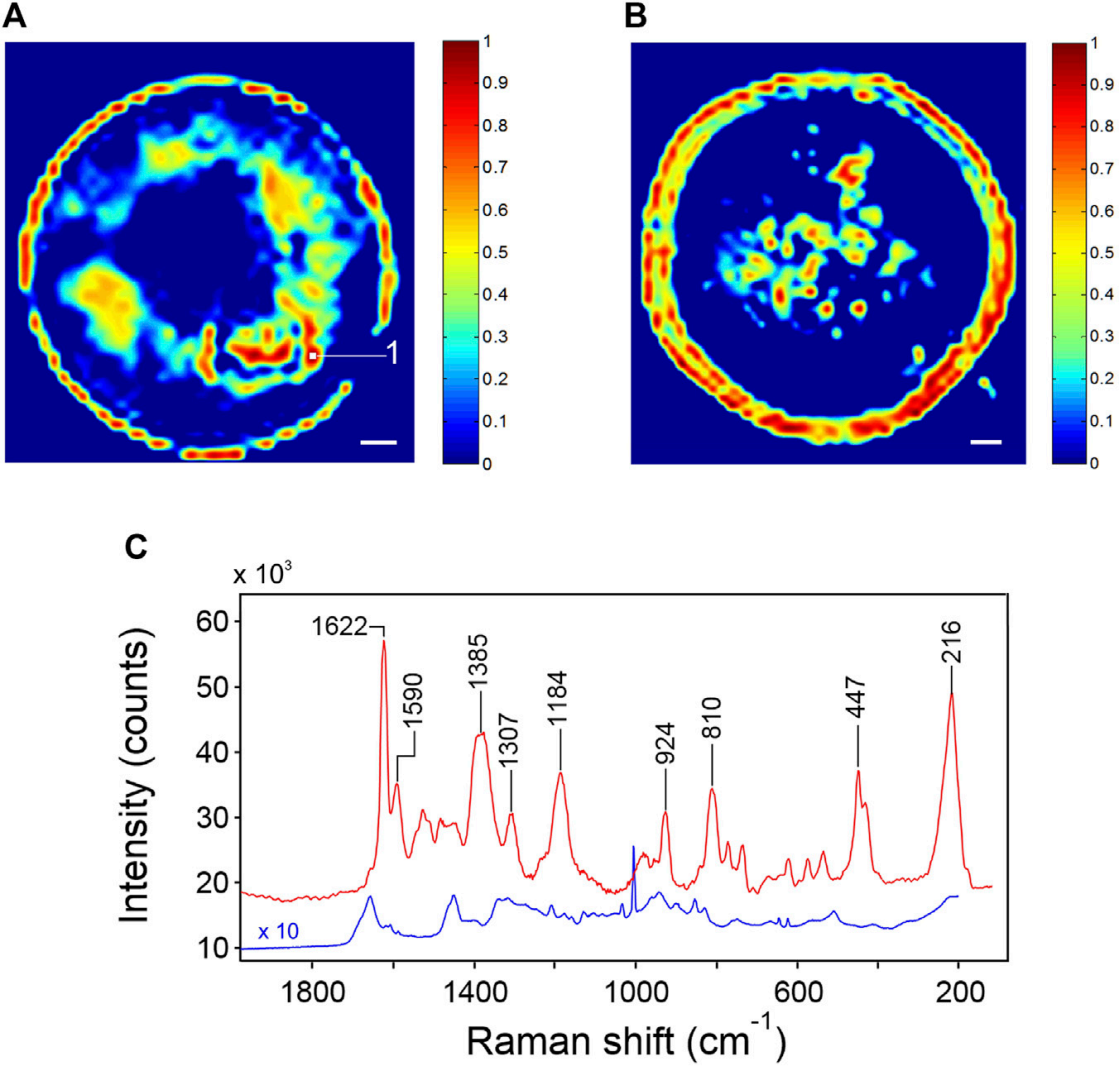
**(A,B)** Raman images of two representative SERS spots functionalized with a 100-μM solution of BSA. The image reconstruction was made by considering the intensity of the 216-cm^−1^ peak. The scale bar is 20 μm. **(C)** SERS spectrum of BSA collected in point one of Figure 7A (red trace); Raman spectrum of solid BSA (blue trace).

The p-jet SERS substrate is characterized here for the first time in case of a typical test protein, the BSA, considering that SERS spectroscopy is a highly promising approach for biosensing (Westermeier and Marouga, 2005; Han et al., 2009; Kuhar et al., 2021). Figures 7A,B show two typical Raman images obtained for the SERS p-jet spots in which a 100-μM solution of BSA was drop-casted. The SERS spectrum collected at a point of maximum activity is shown in Figure 7C, red trace. Image reconstruction was performed by representing the intensity of the peak at 216 cm^−1^ as a function of the collection coordinates. As for the case of R6G, SERS activity is larger in the periphery of the deposition but, especially in the map of Figure 7A, regions of intense signaling are present in the whole area. The round shape of the spots is well defined, confirming the efficiency of the p-jet for the nanostructure confinement. The SERS spectrum of BSA in the areas of high throughput is intense in comparison to previously reported spectra (Das et al., 2009; Iosin et al., 2009; Szekeres and Kneipp, 2018), which denotes a remarkable sensitivity of the proposed platform. The highest EF amounts to 6.6 × 10^5^; the value averaged on the whole sensing area and on five different spots is 2.5 × 10^4^. Also in this case, the platform sensitivity could be further exploited by making the nanoparticle distribution more homogeneous.

In Figure 7C, the SERS spectrum of BSA collected on the present plasmonic platform (red trace) is compared with the Raman spectrum of BSA in the solid state (blue trace). BSA is a weak Raman scatterer: in the solution, the Raman spectrum is barely detectable (Kuhar et al., 2021), but in the dehydrated form, it can be collected with a good SNR and an adequate resolution. The spectrum is reported in full in Supplementary Figure S3, Supplementary Material. As expected, the pattern is complex, reflecting the composition of the protein (583 amino acids, with the aromatic residues accounting for 8.2% of the total). Despite the reduced content, aromatics display numerous signals (e.g., at 1,032, 852, 828, 643, and 622 cm^−1^, among others) in the form of characteristically sharp peaks, owing to the large Raman cross-section of the relatively normal modes. The prominent signal at 1,003 cm^−1^ is due to a breathing mode of the phenyl ring. Broader bands, usually displaying multicomponent structures (e.g., at 1,655, 1,268, and 941 cm^−1^), originate from the amide linkage and reflect its conformational and H-bonding sensitivity. Methylene groups give rise to an intense signal at 1,449 cm^−1^.

The SERS spectrum of BSA deposited on the nanosphere assembly differs substantially from its Raman counterpart. As a preliminary observation, it is noted that the SERS pattern is simpler and considerably more resolved. This effect is likely due to the existence of a preferential interaction site within the protein structure, so that the observed pattern is only representative of the amino acid residue(s) in direct contact with the metal surface. In the SERS spectrum, the main peak occurs at 1,622 cm^−1^: its sharpness, symmetry, and intensity are indicative of a specific functional group in direct contact with the gold surface. BSA displays both negatively charged domains [glutamic or aspartic acid (Glu/Asp), which, at neutral pH, occur in the glutamate/aspartate form] and positively charged domains (lysine or histidine). The nanospheres of the present platform display a positive surface charge (+43 ± 3 mV, as per *z-*potential measurements), suggesting an interaction mechanism through carboxylate ions. In fact, the 1,622 cm^−1^ component has been associated to the antisymmetric stretching of the –COO^−^ group (Aliaga et al., 2009; Chuang and Chen, 2009). The close proximity of the carboxylate anion to the metal surface is confirmed by the substantial intensity of other signals originating from this functional group, at 1,385 cm^−1^ (the COO^−^ symmetric stretching) at 924 cm^−1^ (the C–COO^−^ stretching), and at 447/430 cm^−1^ (out-of-plane deformation modes) (Aliaga et al., 2009).

The well-resolved peak at 1,590 cm^−1^ has been also related to the ν_as_ (COO^−^) mode (Sjöberg et al., 2014) and is tentatively ascribed to the occurrence of an alternative adsorbate conformation. An important band at 1,184 cm^−1^ remains to be elucidated. The intense signal at 216 cm^−1^, entirely absent in the Raman spectrum, has been already reported at close frequencies for a series of amino acids adsorbed on silver colloids (Suh and Moskovits, 1986). It was ascribed to a generic “metal–molecule vibration,” in the present case a COO^−^Au deformation. This band is important for several reasons: it is the second most intense feature of the SERS spectrum and is fully resolved, which makes it an ideal candidate for analytical purposes and imaging reconstruction. It is a direct signature of the analyte-to-metal interaction and, upon a complete vibrational characterization, may provide relevant information on the adsorbate structure. A further notable observation is the absence of aromatic signals in the SERS spectrum (in particular, the lack of the intense peak at 1,004 cm^−1^) and the reduced intensity of the methylene deformation band. This confirms the two non-aromatic residues Glu and Asp as the most likely binding sites and suggests that the carboxylate ion is oriented in such a way to displace the methylene group from the metal surface.

The SERS spectrum of BSA across all the sensing surfaces was remarkably reproducible. This observation, which has relevant implications in biosensing, was quantitatively verified by applying multivariate analysis to the hyperspectral datasets. A principal component analysis (PCA) reduces in a compact form the information broadly distributed in large spectral data; it yields two matrices called scores and loadings that, taken together, reproduce the data variance (Malinowski, 1991). One of the aims of the analysis is to identify the minimum number of linearly independent components (principal components, PC) that is necessary to simulate the dataset. When applied to hyperspectral measurements taken on a mixture, this figure represents the number of individual components contributing to the overall pattern (Martens and Næs, 1989; Windig et al., 2005).

PCA applied to the hyperspectral data of Figure 7A (details of the analysis are reported in Supplementary Figures S4, 5) yielded two significant PCs (PC1 and PC2) accounting for 99.54% of the total variance. Of these, PC1 accounts for 99.17% and PC2 for 0.43% of the total variance. The remaining 0.4% was due to random noise. In practice, a single spectrum (PC1) is able to reproduce the whole data-set composed of 1,935 spectra. Essentially coincident results were obtained for the remaining four spots. This finding is in contrast with those reported in Szekeres and Kneipp (2018) on the SERS response of the protein corona of gold nanoparticles. The spectra collected on colloidal solutions were weak, complex, and displayed large point-to-point variability. These results were interpreted assuming that BSA is unable to substitute the stabilizing citrate layer surrounding the nanoparticles, and the binding occurs *via* electrostatic interaction of the lysine residues with the citrate layer. The complex and poorly resolved SERS patterns were ascribed to an enhanced conformational flexibility of the protein binding sites. A similar behavior was observed for l-lysine adsorbed on silver colloids (Aliaga et al., 2009). In our case, the nanospheres are surrounded by a CTAB bilayer, which is easier to displace than the citrate monolayer (Hunter, 1989; Pannico and Musto, 2021). The consistency of the SERS spectrum, demonstrated by PC analysis, suggests a highly specific orientation/conformational arrangement of the adsorbate, which implies a direct contact of the binding site with the gold surface as a consequence of the bilayer displacement. The reproducible SERS pattern of BSA is the first fundamental step in the direction of future bioanalytical applications.

## 4 Conclusion

In the present contribution, we describe the fabrication of spatially confined SERS surfaces having precisely controlled shape and size, by means of an advanced ink-jet printing technique called pyro-electrohydrodynamic jet printing. This methodology affords the realization of reproducible nanoparticle assemblies with a round shape and a diameter in the 100–200-μm range. This SERS substrate has been developed to optimize single-measurement sampling with current micro-Raman instrumentation. In fact, they improve the platform sensitivity by concentrating the nanospheres in a micro-sized surface and minimize under sampling issues. The SERS activity, tested with R6G was found to be strong and reproducible for a series of five consecutive constructs. Further improvement in the nanoparticle distribution within the sensing area is expected by surface derivatization. The SERS response was also tested for a typical test protein, the BSA, by drop-casting a 100-μM solution of BSA after p-jet spotting. Intense SERS activity was revealed across the whole surface. Furthermore, spectra collected in different locations displayed excellent consistency, as demonstrated by multivariate analysis of the imaging hyperspectral data. The characterization results presented in this work represent the first demonstration of the robustness of this kind of SERS substrate and open the route to future tests for biosensing applications.

## Data Availability

The original contributions presented in the study are included in the article/Supplementary Material, further inquiries can be directed to the corresponding authors.
