# Supplementary material for: Label-Free Protein Analysis by Pyro-Electrohydrodynamic Jet Printing of Gold Nanoparticles
Source: Front Bioeng Biotechnol. 2022 Feb 22;10:817736. doi: 10.3389/fbioe.2022.817736 (PMC8902359; doi:10.3389/fbioe.2022.817736)
Supplement: Supplementary file 1 [file DataSheet1.PDF]

Label-free Protein Analysis by Pyro-electrohydrodynamic jet Printing of Gold Nanoparticles

Veronica Vespini1, Simonetta Grilli[1], Pietro Ferraro[1,*], Romina Rega[1],Heidi Ottevaere[2], Yunfeng Nie[2], Pellegrino Musto[3,*], Marianna Pannico[3]

[1]: Institute of Applied Sciences and Intelligent Systems, National Research Council of Italy (CNR-ISASI), 80078 Pozzuoli, Italy
[2]: Vrije University of Brussels Pleinlaan 2, 1050 Brussels, Belgium
3: Institute for Polymers, Composites and Biomaterials, National Research Council of Italy (CNR-IPCB), 80078 Pozzuoli, Italy

Supplementary Material

Table of contents

1. SIA Analysis pag. 1
2. UV/VIS spectroscopy pag. 1
3. EF calculation for the SERS planar sensor pag. 2
4. Raman spectroscopy pag. 3
5. PC Analysis pag. 3

1. SIA analysis

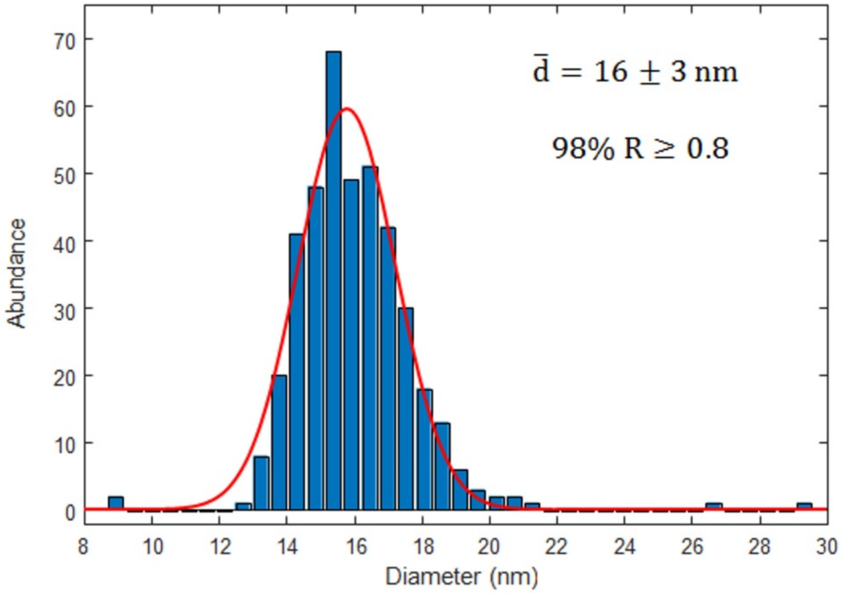

Figure S1. *Statistical Image Analysis of the TEM micrographs.*

2. UV-VIS Spectroscopy

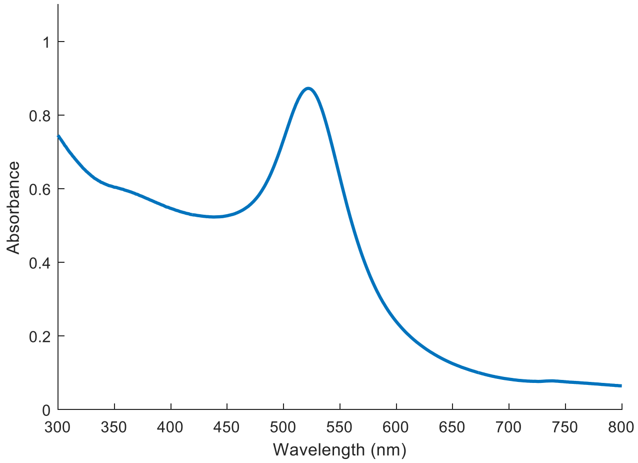

Figure S2. *UV-VIS spectrum of the AuNPs colloid. Au concentration = 2.0 mM. Nanoparticles concentration = 17 nM.*

3. *EF* calculation for the SERS planar sensor

The absolute Enhancement Factor, EF, for the SERS planar sensor was calculated according to:

$$EF = \frac{I_{SERS}}{I_{REF}} \cdot \frac{N_{REF}}{N_{SERS}} \quad (1)$$

The reference Raman spectrum of R6G was collected in solution and the number of scattering molecules (N_{REF}) in the sampling volume was calculated according to:

$$N_{REF} = B_{\upsilon} \times [R6G] \times N_A \quad (2)$$

where $[R6G]$ is the molar concentration of the R6G solution (2.0 mM), N_A is the Avogadro number and B_{υ} is the optical excitation volume. B_{υ} was estimated by determining the transverse dimension (S_{laser} = x · y) and the waist length of the focal volume (z dimension). S_{laser}, was calculated by multiplying the x and y dimensions (radius of the laser) which are determined according to $1.22 \times \frac{\lambda_{laser}}{NA}$ $(eq. 3)$. The z dimension was evaluated by depth profile (*z-profile*) measurements on a silicon wafer. The *z-profile* measurements were carried out by a confocal Raman spectrometer (Labspec Aramis, from Horiba-Jobin Yvon, Edison, NJ, USA) with the following operating conditions: HeNe laser excitation source emitting at 632 nm, metallurgical objective (MPlan 10×, NA = 0.25), confocal and slit apertures set to 400 μm and 1s exposure time. By varying the focus of the laser along the z axis the intensity variation of the silicon peak (520 cm^{-1}) is a bell-shaped function from which the Full Width at Half Maximum (FWHM) gives the waist length of the focal volume (z dimension). The resulting B_{υ} is 2.16×10^{-9} cm^3 and the resulted N_{REF} is 2.73×10^9.

The number of molecules contributing to the SERS effect (N_{SERS}) on the planar surface was estimated by the ratio of the total metal surface illuminated by the laser beam ($S_{laser} = 9.51 \times 10^6$ nm^2, calculated according to eq.3) and the geometrical cross section of the individual R6G molecules (4.0 nm^2). The calculated N_{SERS} is 2.38×10^6.

4. Raman spectroscopy

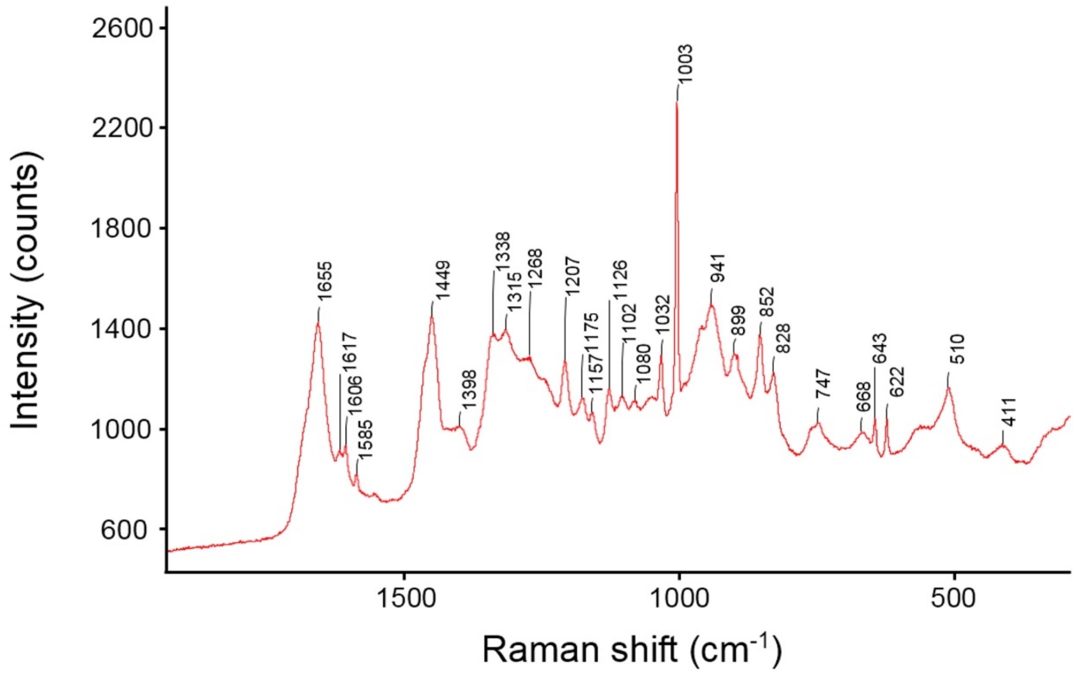

Figure S3. *Raman spectrum of solid BSA.*

5. PC Analysis

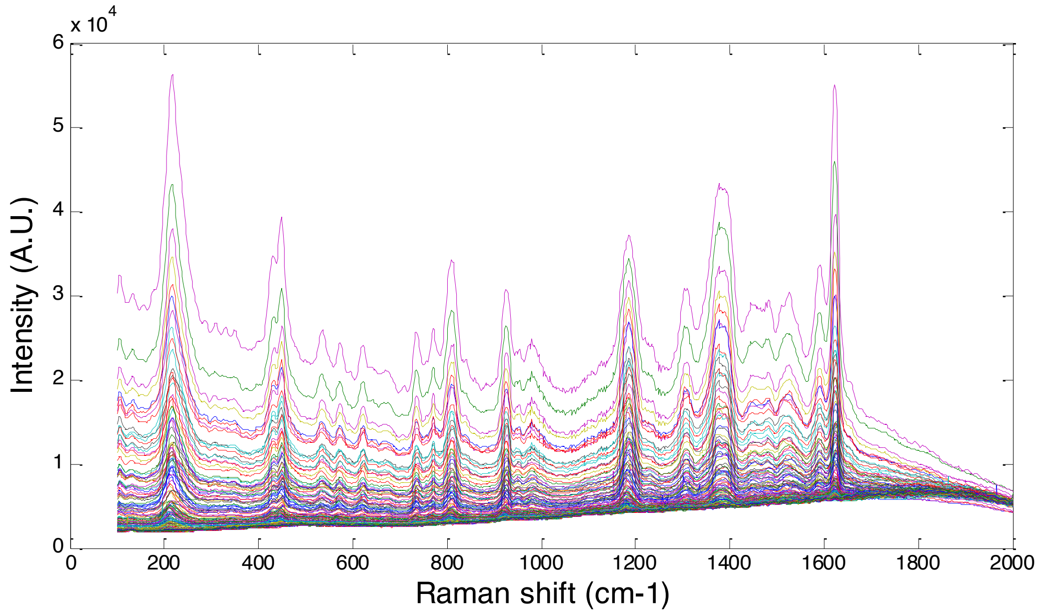

Figure S4. *Hyperspectral data set for the Raman image of Fig. 7A.*

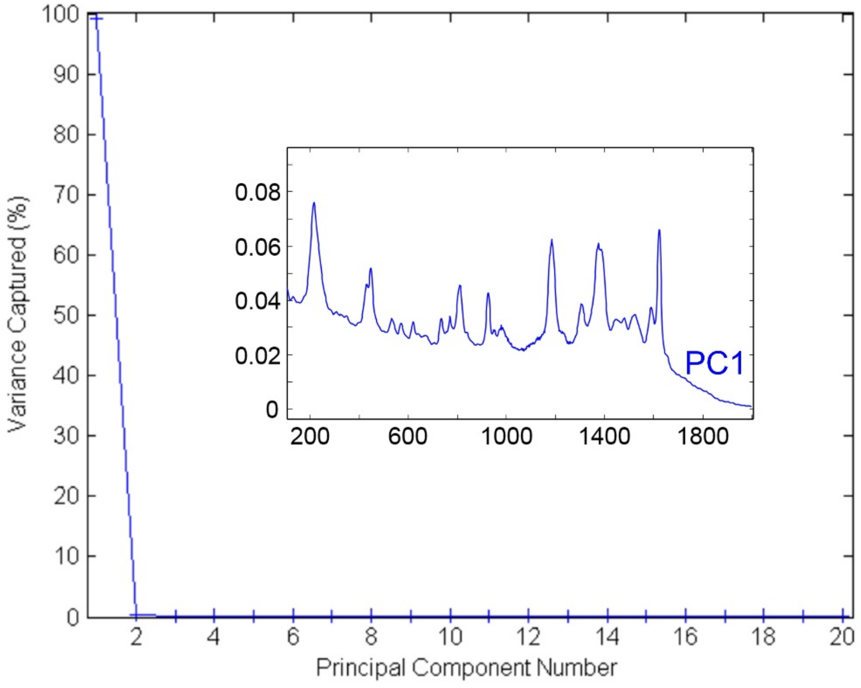

Figure S5. *Percent of variance captured by the principal components identified by PCA. The inset represent the PC1 component.*
